# Understanding the Influence of Individual and Systemic Factors on Vaccination Take-Up in European Citizens Aged 55 or Older

**DOI:** 10.3390/vaccines9020169

**Published:** 2021-02-17

**Authors:** Olympia E. Anastasiou, Dörte Heger

**Affiliations:** 1 Institute for Virology, University Hospital Essen, University of Duisburg-Essen, 45147 Essen, Germany; 2 RWI–Leibniz Institute for Economic Research, 45128 Essen, Germany; doerte.heger@rwi-essen.de

**Keywords:** vaccination, vaccination hesitancy, vaccine knowledge

## Abstract

*Background:* High vaccination coverage provides extensive public health benefits. Hence, increasing vaccination rates is an important policy goal within the EU and worldwide. We aim to evaluate individual and systemic parameters associated with vaccination in European Union citizens aged 55 or older, using data from the Special Eurobarometer 488. *Methods:* Linear probability and probit models are estimated to analyze the determinants of vaccination take-up. Further, descriptive analyses are used to explore how the reasons for not having a vaccination differ by welfare regime. *Results:* High knowledge about the effectiveness and safety of vaccination increases the probability of receiving a vaccination during the past five years by 26 percentage points (pp), medium knowledge increases it by 15 pp. Focusing on the specific case of the flu, official recommendations increase this probability by, on average, 6 pp; while having to pay out-of-pocket for a recommended vaccination decreases it by, on average, 10 pp. Furthermore, the differences for no vaccination differ widely across welfare systems and television is the primary source for information about vaccination. *Conclusions:* Reported vaccination rates in Europe fall far below targets set by official recommendations. Increasing vaccination knowledge and offering vaccinations free of charge can help to increase vaccination rates. A specific focus should be put on reaching individuals with potential difficulties of access such as those living alone and unemployed.

## 1. Introduction

Vaccination is one of the most effective tools of preventive medicine and one of the greatest medical achievements of the last century [1]. According to the WHO, 2–3 million deaths per year are prevented due to vaccination and another 1.5 million deaths could be prevented by improving global vaccination coverage. Vaccine hesitancy, defined as “the reluctance or refusal to vaccinate despite the availability of vaccines” is included in the “Ten threats to global health in 2019” report of the WHO [2]. Vaccinations are recommended by the European Centre for Disease Prevention and Control (ECDC) and various national and international medical societies, even when the vaccination strategies vary by a certain degree in different countries of the European Union (EU) [3]. The benefits of vaccination for the public health have been demonstrated repeatedly in numerous high-quality studies [4,5]. In the scientific community and in the public sphere vaccines are generally viewed as both effective and safe. Nevertheless, there is a growing number of groups and individuals expressing concerns about vaccines that delay or refuse the vaccination of themselves and/or their children [4]. The reasons underlying the issue are numerous including individual and group-related influences, as well as health system and vaccine-associated parameters, varying significantly amongst different populations [4,6]. 

Even if vaccination is often linked to children as a target group in the media, it is nevertheless also crucial for the elderly population. Senior citizens make up for an increasing part of the population in high income countries. Due to immunosenescence and frequent comorbidities, they have an increased susceptibility for infections and are at a higher risk for infectious disease-related morbidity and mortality [7]. Vaccine-preventable diseases cause a substantial human and economic burden [8]. Vaccination is an effective strategy to prevent such outcomes and reduce health-related costs; however, vaccination coverage tends to be consistently lower for adults compared to children [9]. Thus, it is relevant both from a clinical and an economic point of view to scrutinize factors associated with vaccine hesitancy in this population group in order to generate more efficient vaccination strategies.

The aim of the present study is to evaluate parameters, both individual and systemic, associated with vaccination in European Union citizens aged 55 or older. In more detail, we use a linear probability model to analyze the impact of various socio-demographic characteristics such as sex, age, educational level, living situation, and income as well as characteristics of the health care system including the type of welfare regime and official vaccination recommendations. Further, we explore in descriptive analyses how the reasons for not having a vaccination differ by welfare regime.

## 2. Methods

### 2.1. Data

The analysis in this study was conducted with data derived from the Special Eurobarometer 488 (Wave EB91.2) “Europeans’ attitudes towards vaccination”, which collected data on the attitude of European Union citizens on vaccines and vaccination in 2019. The Eurobarometer program included a series of cross-national studies designed to evaluate and compare trends within Europe. This survey was carried out by the Kantar network in the 28 Member States with 27,524 respondents aged 15 or older being interviewed at home face-to-face in their native language. Sampling was performed with a multistage random probability approach, including ca. 1000 respondents from each participating country with the exception of Cyprus, Luxembourg and Malta, where ca. 500 respondents were interviewed [10]. Data from the Eurobarometer survey series are publicly accessible. 

For the analysis of this study, the sample is restricted to adults aged 55 or older, leaving 13,082 individuals. After removing 279 (2.1%) cases with missing values, our final sample included 12,803 individuals.

### 2.2. Variables

Our main variable of interest is whether an individual has received a vaccination within the past five years (QC3, code 1). 

As explanatory variables we follow Andersen’s model of health care utilization and include predisposing, need, and enabling variables. Andersen’s model of health care utilization is a conceptual model, whose aim is to describe the factors that determine the utilization of health services. Contextual and individual characteristics combine to modify health behaviors and ultimately health outcomes. The factors are grouped in the following categories: predisposing (e.g., age, country of origin), enabling (e.g., individual financial situation, health policy) and need-related (e.g., mortality rate of a particular disease, personal perceived needs) [11]. More detail about the individual as well as systemic variables used and the vaccination schedule for the general population 55+ based on the ECDC vaccine scheduler can be found in the Supplementary Materials in Text 1 and Table S1, respectively. In this study, we focus on influenza vaccination according to the general recommendation for both practical and public health reasons. Since it is yearly recommended and the most frequently applied vaccination, presumably generating the majority of positive answers to the question of vaccination, we use the recommendation for influenza vaccination in a given population as a measure that having had a vaccination within the past five years is needed. 

While almost all countries recommend a flu vaccination for their population from a certain age on, the vaccination is not always covered. Since having to pay for the vaccination imposes a possible barrier to access, we include an indicator for whether the flu vaccination is not covered despite being recommended as enabling variable. Further enabling variables are financial hardship, an indicator that measures whether the respondent has had difficulty paying bills within the last year at least from time to time, whether the respondent is retired, whether the respondent is still working (the reference categories include the unemployed, temporary unable to work, and house persons), and the respondent’s education level. The latter variable includes three categories: low education (education ended before age 15, which serves as reference category), medium education (education lasted until age 16–19), and high education (education lasted until age 20 or longer). Respondents who stated they are still studying where excluded from the analysis.

As predisposing variables, we include age as linear, quadratic, and cubic term, as well as indicators for being female, living alone (single household), and the country of residence. Further, we use knowledge about the effectiveness and safety of vaccines as a measure for health beliefs (vaccination knowledge). The variable is based on whether the respondent agrees (true vs false) with the four statements “Vaccines overload and weaken the immune system”, “Vaccines can cause the disease against which they protect”, “Vaccines can often produce serious side-effects”, “Vaccines are rigorously tested before being authorized for use”, where only the last statement is true. The indicator “High vaccination knowledge” equals one if the respondent has answered at least two questions correctly. “Medium knowledge” requires at least one correctly answered question.

Further country characteristics like general trust in authorities [12], national vaccination campaigns [5,13] or doctors’ quality [14] might play a large role in determining vaccination behavior and are captured by the inclusion of country dummies. In addition, we are interested whether more general aspects like the organization of the health care system influences vaccination uptake. For this reason, we control for different welfare systems. We group countries based on the model by Esping-Andersen (1990) [15] and extended by Fenger (2007) [16] into the categories “post-communist” (Bulgaria, Croatia, Czech Republic, Hungary, Poland, Romania, Slovakia, and Slovenia), “former USSR” (Estonia, Latvia, and Lithuania), “Anglo-Saxon/liberal” (Ireland and the United Kingdom), “conservative/corporatist” (Austria, Belgium, Cyprus, France, Germany, Greece, Italy, Luxembourg, Malta, the Netherlands, Portugal, and Spain), and “social-democrat/Nordic” (Denmark, Finland, and Sweden). Though Romania has originally been categorized as “developing”, we include the country within the post-communist countries since only one country in our sample would fall into this category. A dummy variable for each group is included in the model. The group of post-communist countries serves as reference category. Moreover, one country per group has to be excluded as reference category. We excluded France, Great Britain, Sweden, Latvia, and Hungary.

### 2.3. Statistical Analysis

Simple means by country and age groups were calculated to report population vaccination rates. Post-stratification weights were used for the descriptive analysis to produce nationally representative estimates.

To estimate the effect of the explanatory variables on the probability of having had a vaccination during the past five years a linear probability model (LPM) was used. Besides our main estimation, we estimate the model (i) using clustered standard errors by country, (ii) excluding the two countries with mandatory vaccinations (Bulgaria and the Czech Republic, see Supplementary Materials), (iii) excluding countries that recommend the flu vaccination for all individuals 55+ (Austria, Malta, Poland), and (iv) using a probit model to explicitly account for the binary nature or our dependent variable as robustness checks. We exclude the selected countries in specification (ii) and (iii) since our indicator for the flu recommendation might be less informative if individuals received a mandatory vaccination within the past five years or when the recommendation applies to all individuals in a country. No weighting has been used in the multivariate analyses. Instead, the estimates are intended to represent the effects for the included sample. All analyses were performed using Stata SE 14 (StataCorp., College Station, TX, United States). 

## 3. Results

### 3.1. Descriptive Results

Table 1 reports weighted vaccination rates by age group and by whether or not the flu vaccination is recommended for all countries in our sample. In most cases, vaccination rates increase by age. In addition, groups with a general flu vaccination recommendation tend to have higher vaccination rates compared to those without. However, some countries have relatively low (e.g., Bulgaria and Poland) or relatively high (e.g., Germany and Finland) vaccination rates across all age groups. Furthermore, in Bulgaria, Slovakia, and Latvia the vaccination rates for those who are recommended to have a flu vaccination are lower than for those without such a recommendation, though this difference is only statistically significant in Latvia.

Descriptive statistics of our sample are shown in Table 2: 46% of respondents report that they have received a vaccination within the past five years, compared to 67% for which the flu vaccination has been recommended based on their age and country. Since Table 2 shows unweighted sample means, the numbers differ slightly from the population averages presented in Table 1. While the flu vaccination is mostly covered, this has not been the case for 12% of the respondents. Slightly less than half of the study population (45%) could answer at least one of the four questions about vaccinations correctly and is referred to as having medium vaccination knowledge, 43% have high knowledge. The sample includes slightly more women (55%), the mean age lies just below 68 years and 33% live in single person households. Most respondents fall into the category medium education (44%), while 25% and 31% belong to the category low and high education, respectively. Approximately two thirds of the sample (67%) are retired, while 27% are still working. Almost a third (27%) has experienced financial hardship during the past year. The countries are represented relatively equally with 1.7% of the sample coming from Luxembourg and 6.2% from Germany, 25% fall within the group of post-communist countries, 13% are the former USSR countries, 7% are Anglo-Saxon/liberal countries, 41% are conservative/corporatist countries, and the remaining 14% are social-democrat/Nordic countries.

### 3.2. Explanatory Factors of Vaccination Up-Take

Estimation results are shown in Table 3. On average, a recommendation for the flu vaccination increases vaccination rates by 6 percentage points (pp). Using the Europe-wide share of individuals who have received a vaccination within the past five years (48%) as baseline, this corresponds to a 13% increase. If the recommended flu vaccination is not covered by the health care systems, average vaccination rates are approximately 10 pp lower (8 pp lower in model (3), which excludes Bulgaria where the flu vaccination is not covered). These results are highly statistically significant and largely robust throughout our different specifications. The probit model provides very similar though slightly lower estimates with a 5 pp increase and 9 pp decrease of average vaccination rates given a flu recommendation and the flu vaccination not being covered, respectively. Medium and high knowledge about vaccinations increases vaccination rates by 15 pp and 26 pp, respectively across models (1) to (4) and by 16 pp and 27 pp in the probit model. That is, high vaccination knowledge increases the baseline vaccination rate by 54%. Again, all estimates are highly statistically significant at the 1% significance level.

Further factors increasing or decreasing vaccination take-up are discussed for our main specification only since differences across the various specifications are small. Enabling factors such as high education (+3 pp; *p* < 0.01), being retired (+5 pp; *p* < 0.01), working (+5 pp; *p* < 0.01) increase the probability of having had a vaccination during the past five years, while having difficulties paying bills (−4 pp; *p* < 0.01) reduces this probability. The results for predisposing factors are mixed. Average vaccination rates do not differ by gender. With respect to age, vaccination rates, on average, increase with age between ages 58 and 85, i.e., for most of our considered age range. Compared to the reference category (post-communist countries), vaccination take-up is on average higher in Anglo-Saxon/liberal countries (+20 pp; *p* < 0.01), conservative/corporatist countries (+22 pp; *p* < 0.01), and social-democrat/Nordic countries (+25 pp; *p* < 0.01). In countries formerly belonging to the USSR, average vaccination rates are slightly lower (−6 pp; *p* < 0.1), though the difference is only weakly statistically significant. Further differences exist across countries. However, since the country dummies pick up any remaining differences such as preferences and attitudes towards vaccination or preventive health care use in general, characteristics of the national health care systems, national vaccination campaigns, we cannot interpret their magnitude beyond stating differences in vaccination rates as documented in Table 1 (country estimates available upon request). 

### 3.3. Reasons for no Vaccination

**Figure 1:**
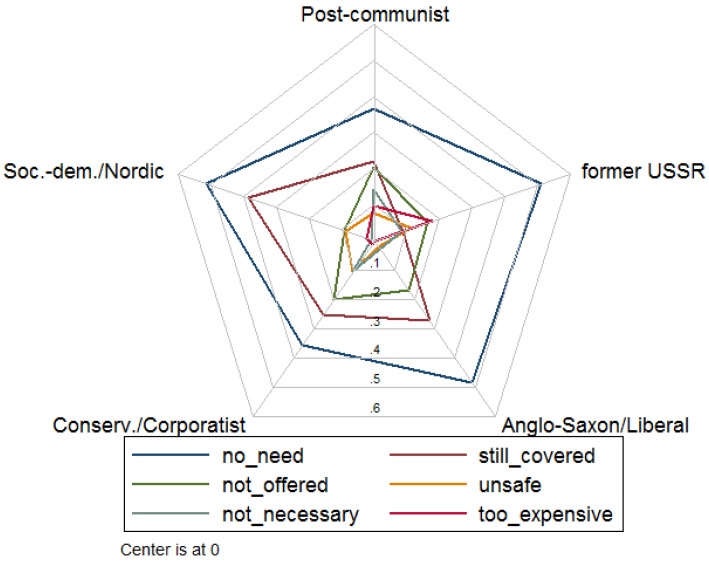
Reasons for no vaccination within the last five years by type of welfare system.

Figure 1 reports the reasons why individuals did not receive a vaccination within the past five years for the groups of countries based on the characteristics of their welfare systems. The categories include “do not see the need to be vaccinated” (no_need), “still covered by vaccines received earlier” (still_covered), the respondent has “not been offered any vaccine” (not_offered) by his or her general practitioner or doctor, “vaccines are not safe and they can have side-effects” (unsafe), “vaccines are only necessary for children” (not_necessary), and “it is expensive” (too_expensive). Additional categories not reported due to low numbers include “it is complicated and requires a lot of effort”, “other”, “no reason”, and “don’t know”. Multiple answers were allowed. Not seeing the need to be vaccinated is the most frequently stated reason in all country groups. In the group of social-democratic/Nordic as well as the former USSR countries, this answer is provided by over 50% of individuals without vaccination. However, it should be kept in mind that the share of not vaccinated individuals is much lower in, for example, social-democratic/Nordic countries (36%) than in post-communist (77%) and the former USSR countries (72%). In all country groups except the former USSR, the second most frequently stated reason is “still covered”. The other reasons are generally less important, yet about 20% state that a vaccination has not been offered in the group of conservative/corporatist countries and post-communist countries. In the former USSR countries, also about 20% state that a vaccination is too expensive.

### 3.4. Information on Vaccination and Attitude on Vaccination Programs

Most individuals in Europe have a positive attitude towards vaccination programs, with only 8.5% rejecting them as shown in Table 4, with 41% of the population 55+ thinking that they should be coordinated at a national level. The percentage of individuals rejecting vaccination programs is highest in the former USSR welfare system (15%) and lowest in the Anglo-Saxon one (3%). A third (34%) of the respondents did not see, read or hear any information on vaccination in the media in the past six months. For those who did, television (56%), followed by the printed press (20%) and the radio (15%), were the sources of information. Internet sources, sites (4%) or social networks (4%), played a minor role. 

## 4. Discussion

The self-reported vaccination rates among European Union citizens (aged 55 or older) within the last five years are generally low, ranging from 15% for Bulgaria to 69% for Finland. These vaccination rates—as depicted in our results—fall considerably short of the target in most European countries. Moreover, taking into account that many countries also recommend other (though less frequent) vaccinations besides the flu vaccination, that more frequent vaccination is recommended for high-risk populations (vs the general population) and that a single vaccination within the last five years leads to the categorization of a participant in the “vaccinated” group, our results provide an upper bound for the vaccination coverage according to vaccination recommendations. For example, an individual for whom the flu vaccination is recommended would be considered “vaccinated” in our analysis if he or she received one vaccination within the past five years even though the flu vaccination is recommended yearly. Thus, our results cannot be taken as the exact overall vaccination coverage per vaccination recommendation. Nevertheless, they provide us with an approximate idea of the vaccination rate, especially when comparing different populations and are, indeed, in line with those from a previous ECDC report. Influenza vaccination coverage rates ranged according to this report from about 2.4% in Latvia to 70% in the UK in respondents older than 65 years [17].

Following the Andersen’s model of health care utilization, we included predisposing (age, sex, single household, country of residence, vaccination knowledge), need (recommendation for flu vaccination), and enabling (flu vaccination reimbursement, work/retirement status, financial hardship, education level) variables to analyze and interpret our data. Focusing on the predisposing factors, we found that older age and high vaccination knowledge are associated with increased vaccination, while the opposite effect is true for living alone and in the presence of financial hardship. Our results are largely consistent with those from an analysis of the self-reported flu vaccination rate in the elderly as measured by the first and second wave of the Survey of Health, Ageing and Retirement in Europe (SHARE) in 2004/2005 and 2006/2007. Full-time work and older age in the 65+ European population group were then positively associated with increased vaccination rate, while the effect of sex was present but minimal [14]. Increased vaccination with age [18,19] and the beneficial effect on vaccination uptake of living with others for the elderly population have also been demonstrated in previous studies [20]. Lack of knowledge or false perceptions about vaccines among the elderly and also healthcare professionals have been often cited as a significant contributor to vaccine hesitancy [4,19,21], in line with our data. On the other hand, we did not have a measurable impact of sex on vaccination rate. Previous studies have generated discrepant results on the association (or lack thereof) of sex and vaccine uptake [22]. For example, female sex has been associated with an improved vaccination rate, as shown in the Swiss study for a measles, mumps and rubella (MMR) vaccination among young adults [23], but also with increased vaccine hesitancy for the vaccination of children as shown in a recent French study [24]. An aspect partly addressed in our study is the role of health beliefs (vaccination knowledge) and experience (financial barriers to flu vaccination) in shaping vaccination hesitancy and thus vaccination rates. According to the health belief model by Rosenstock, perceived susceptibility and severity as well as perceived benefits and barriers combine to modify health related-behavior. The model has been successfully applied with the aim of understanding and predicting vaccination behavior [25,26]. Previous data have indicated that perceived barriers are often the most significant predictors of preventive health behaviors, an observation in accordance with our results since both the lack of flu vaccination reimbursement and the perception of financial hardship were significant predictors of the vaccination rate [27,28]. Although the model is not applicable to our study due to lack of data pertaining to key aspects of the model, it is an approach worth pursuing in future studies with the goal of increasing vaccination rates.

Focusing on enabling variables, we find that flu vaccination reimbursement, participation in the workforce or retirement, lack of financial hardship and high education level are associated with increased vaccination rates. Indeed, perceived financial hardship, deprivation and lower education have been described as having the opposite effect in different populations [18,21,24,29,30,31], while vaccination reimbursement has been linked to increased vaccination in the past based on data from various countries [22,32,33,34]. Last but not least, we find that the existence of recommendation for flu vaccination for the general population has also a positive impact on the vaccination rate. Both latter observations are significant for the public health and reinforce the results of a previous study linking increased vaccination among the elderly with the monitoring of vaccination rates and the distribution of personal letters or vouchers for free vaccination [35]. 

As shown in Table 4, beliefs on whether vaccination program should be established at an (inter)national level or not at all differs largely by different welfare systems. For this reason, we also considered differences in vaccination rates by welfare systems and found considerably higher probability of receiving a vaccination within the past five years in Anglo-Saxon/liberal, conservative/corporatist, and social-democrat/Nordic countries, i.e., in countries with larger support for vaccination programs. 

An important aspect of this study pertains also to the reasons given by participants for non-vaccination. Not seeing the need to be vaccinated is the most frequently stated reason in all country groups. In all country groups except the former USSR, the second most frequently stated reason is “still covered”. Given that for more than two thirds of our sample the flu vaccination is recommended and that other vaccinations are also commonly recommended for the considered age group, the belief to be covered and not needing a vaccination seems puzzling. However, this finding can explain the large influence of knowledge about vaccinations on vaccination rates. Encouragingly, stating safety concerns as the driving force for non-vaccination was very low (ranging from 2% to 11%) in all welfare systems. Previous studies have indicated that seeing no need for vaccination, concerns about vaccine effectiveness and side effects (29%) are frequently given reasons for not vaccination, but the percentage of participants ascribing to the one or other category can differ significantly in different populations [36,37,38].

Since vaccination knowledge seems to be the key in increasing vaccination rates, it is important to understand how individuals acquire such knowledge. Information on vaccination, although readily and universally available in theory, reaches individuals only to a limited degree. Every third respondent reports not receiving any information on vaccination from the media in the past six months. This is not an insignificant percentage, if one considers the annual character of influenza vaccination. Television is the primary medium for information about vaccines, since more than half of the respondents that did get information about vaccination did so through it. On the other hand, the internet (websites or social networks) played reportedly only a minor role. The disparity on the different media abilities to propagate vaccination information should be taken into account when designing an information campaign about vaccination. 

Finally, our study has several strengths and limitations. First, we analyze vaccination behavior of a large sample of respondents across several countries with different welfare systems. Second, the inclusion of both individual and systemic determinants of vaccination enabled us to present a more nuanced picture of the phenomenon than focusing on the former or latter alone would allow. Third, identifying different parameters with a positive or negative effect on vaccination uptake could allow for the planning of more efficient vaccination strategies and guide policy recommendations. Fourth, our study focuses on the very important issue of vaccination uptake and vaccination hesitancy, which becomes especially relevant in the view of the current COVID-19 pandemic. Yet, some limitations remain. Due to its cross-sectional design, it is not possible to further strengthen the appropriateness of the causal interpretation of our results by comparing changes in vaccination recommendations on the individual level. Further, as with most surveys, there is the possibility of reporting bias (recall or social desirability bias). It should also be emphasized that an optimal strategy to increase vaccination rates needs to take account of individual countries´ cultural and social characteristics that go beyond the type of welfare system. The design of our study and the number of participants does not allow for a detailed analysis of the above-mentioned aspects on a national level. These aspects should be addressed in future studies. Last but not least, the available data did not include information on health insurance or medical history (comorbidities), limiting our ability to evaluate their impact on vaccination. Nevertheless, we believe that despite its limitations, our study provides important information on a current and highly relevant for the public health topic.

## 5. Conclusions

To conclude, since vaccination rates in Europe are much lower than the targets set by official recommendations, increasing vaccination rates would be desirable. Some determinants of vaccination cannot be modified in the short term or at all for a given population; others, however, can be. We have demonstrated that knowledge about the effectiveness and safety of vaccines, vaccination recommendation, and reimbursement are significant factors in determining vaccination uptake and all can be addressed as a matter of public health both at a national or even international level. Our data suggest that improving knowledge about the effectiveness and safety of vaccinations and offering vaccinations free of charge would help to increase vaccination rates. Providing information on vaccination via various media outlets might be a promising way to achieve this goal, but also focusing on specific media to reach specific groups is important to increase efficiency. Since vaccination knowledge as well as vaccination rates differ by population groups, information campaigns should put special attention on reaching individuals with potential difficulties of access to the health care system such as those living alone and unemployed. 

## Figures and Tables

**Table 1:** Share of individuals who received a vaccination within the last five years.

	Age					Flu Vaccination		
	55–59	60–64	65–69	70–74	75+	not rec.	rec.	All
Conservative/Corporatist								0.53
Austria	0.43	0.43	0.44	0.48	0.61	-	0.48	0.48
Belgium ***	0.45	0.47	0.48	0.71	0.64	0.46	0.60	0.55
Cyprus ***	0.26	0.13	0.29	0.40	0.57	0.19	0.42	0.33
France ***	0.41	0.50	0.50	0.66	0.70	0.46	0.61	0.55
Germany	0.62	0.64	0.70	0.65	0.70	0.62	0.68	0.67
Greece	0.25	0.26	0.52	0.62	0.76	0.25	0.56	0.51
Italy	0.26	0.28	0.27	0.44	0.30	0.27	0.33	0.31
Luxembourg	0.61	0.54	0.72	0.59	0.69	0.56	0.67	0.63
Malta	0.34	0.51	0.60	0.55	0.70	-	0.54	0.54
Netherlands **	0.46	0.58	0.59	0.54	0.57	0.46	0.57	0.55
Portugal **	0.55	0.59	0.71	0.66	0.69	0.57	0.69	0.64
Spain ***	0.31	0.38	0.55	0.60	0.74	0.35	0.65	0.54
Social-democrat/Nordic								0.64
Denmark **	0.44	0.48	0.57	0.59	0.59	0.46	0.58	0.54
Finland	0.70	0.64	0.70	0.78	0.68	0.67	0.71	0.69
Sweden ***	0.47	0.57	0.73	0.75	0.77	0.52	0.76	0.67
Anglo-Saxon/Liberal								0.57
Ireland ***	0.34	0.47	0.62	0.64	0.60	0.40	0.62	0.53
United Kingdom ***	0.31	0.43	0.60	0.67	0.74	0.37	0.69	0.57
Post-communist								0.23
Bulgaria	0.18	0.15	0.12	0.15	0.16	0.17	0.14	0.15
Croatia	0.29	0.20	0.28	0.42	0.40	0.24	0.35	0.31
Czech Republic	0.46	0.36	0.47	0.42	0.44	0.41	0.44	0.43
Hungary **	0.14	0.21	0.20	0.29	0.38	0.14	0.26	0.23
Poland	0.20	0.13	0.16	0.18	0.15	-	0.16	0.16
Romania **	0.08	0.22	0.20	0.24	0.30	0.15	0.24	0.20
Slovakia	0.38	0.33	0.35	0.39	0.40	0.38	0.37	0.37
Slovenia *	0.32	0.26	0.35	0.42	0.38	0.29	0.37	0.34
Former USSR								0.28
Estonia	0.36	0.21	0.29	0.25	0.30	0.28	0.29	0.29
Latvia **	0.43	0.43	0.34	0.29	-	0.43	0.31	0.36
Lithuania	0.20	0.18	0.26	0.28	0.24	0.23	-	0.23
Total	0.38	0.40	0.46	0.54	0.60	0.39	0.51	0.48

Note: Vaccination rates are weighted to represent the population 55+. ***, **, and * denote significant differences in vaccination rates by recommendation status at the 1%, 5%, and 10% level, respectively.

**Table 2:** Descriptive statistics.

	Mean	Std. Dev.	Min	Max
Vaccinated	0.462	0.499	0	1
Predisposing factors (Andersen’s model of health care utilization)				
Medium vaccination knowledge	0.448	0.497	0	1
High vaccination knowledge	0.432	0.495	0	1
Female	0.546	0.498	0	1
Age	67.968	8.469	55	98
Single household	0.328	0.470	0	1
Welfare system				
post-communist	0.252	0.434	0	1
former USSR	0.127	0.333	0	1
Anglo-Saxon/Liberal	0.072	0.258	0	1
Conservative/Corporatist	0.410	0.492	0	1
Social-democrat/Nordic	0.139	0.346	0	1
Country of Residence				
Austria	0.027	0.161	0	1
Belgium	0.037	0.188	0	1
Bulgaria	0.034	0.181	0	1
Croatia	0.024	0.153	0	1
Cyprus	0.020	0.139	0	1
Czech Republic	0.028	0.165	0	1
Denmark	0.042	0.201	0	1
Estonia	0.046	0.209	0	1
Finland	0.048	0.215	0	1
France	0.039	0.193	0	1
Germany	0.060	0.238	0	1
Greece	0.036	0.186	0	1
Hungary	0.034	0.180	0	1
Ireland	0.031	0.172	0	1
Italy	0.031	0.172	0	1
Latvia	0.037	0.188	0	1
Lithuania	0.045	0.207	0	1
Luxembourg	0.017	0.129	0	1
Malta	0.024	0.153	0	1
Netherlands	0.052	0.223	0	1
Poland	0.033	0.178	0	1
Portugal	0.036	0.186	0	1
Romania	0.026	0.160	0	1
Slovakia	0.036	0.187	0	1
Slovenia	0.038	0.191	0	1
Spain	0.033	0.178	0	1
Sweden	0.048	0.214	0	1
United Kingdom	0.041	0.198	0	1
Enabling factors (Andersen’s model of health care utilization)				
Education				
Low (<age 15)	0.249	0.432	0	1
Medium (age 16–19)	0.438	0.496	0	1
High (age 20+)	0.313	0.464	0	1
Retired	0.671	0.470	0	1
Working	0.265	0.442	0	1
Financial hardship	0.272	0.445	0	1
Flu vaccination recommended but not covered	0.124	0.329	0	1
Need factor (Andersen’s model of health care utilization)				
Flu vaccination recommended	0.667	0.471	0	1

Note: Unweighted sample statistics. N = 12,803.

**Table 3:** Estimation results.

	(1)	(2)	(3)	(4)	(5)
Dependent variable:	LPM	LPM	LPM	LPM	Probit
vaccinated within the last 5 years	(robust std. err.)	(clustered std. err.)	(w/o BU, CZ)	(w/o AT, MT, PL)	(marginal effects)
Flu vacc. recommended	0.059 ***	0.059 **	0.056 ***	0.057 ***	0.050 ***
	(0.016)	(0.024)	(0.017)	(0.018)	(0.016)
Flu vacc. not covered	−0.097 ***	−0.097 ***	−0.082 ***	−0.097 ***	−0.086 ***
	(0.025)	(0.027)	(0.031)	(0.025)	(0.030)
Medium vacc. knowledge	0.149 ***	0.149 ***	0.154 ***	0.151 ***	0.164 ***
	(0.012)	(0.018)	(0.013)	(0.013)	(0.014)
High vacc. knowledge	0.258 ***	0.258 ***	0.263 ***	0.264 ***	0.269 ***
	(0.013)	(0.020)	(0.013)	(0.013)	(0.014)
Female	0.002	0.002	−0.001	−0.001	0.004
	(0.008)	(0.010)	(0.009)	(0.009)	(0.008)
Age	−0.200 ***	−0.200 **	−0.190 ***	−0.187 **	−0.197 ***
	(0.071)	(0.074)	(0.073)	(0.074)	(0.071)
Age^2^/100	0.290 ***	0.290 **	0.278 ***	0.273 ***	0.286 ***
	(0.099)	(0.105)	(0.102)	(0.103)	(0.099)
Age^3^/10,000	−0.135 ***	−0.135 **	−0.130 ***	−0.128 ***	−0.134 ***
	(0.046)	(0.049)	(0.047)	(0.047)	(0.046)
Single household	−0.031 ***	−0.031 ***	−0.031 ***	−0.027 ***	−0.031 ***
	(0.009)	(0.010)	(0.010)	(0.010)	(0.009)
Low education	−0.024 **	−0.024	−0.028 **	−0.028 **	−0.024 **
	(0.011)	(0.015)	(0.012)	(0.012)	(0.011)
High education	0.031 **	0.031 *	0.026 **	0.025 *	0.031 **
	(0.013)	(0.018)	(0.013)	(0.013)	(0.013)
Retired	0.054 ***	0.054 ***	0.057 ***	0.058 ***	0.055 ***
	(0.018)	(0.016)	(0.019)	(0.020)	(0.019)
Working	0.049 ***	0.049 **	0.048 **	0.053 ***	0.051 ***
	(0.019)	(0.018)	(0.019)	(0.020)	(0.019)
Financial hardship	−0.042 ***	−0.042 ***	−0.042 ***	−0.037 ***	−0.045 ***
	(0.011)	(0.011)	(0.011)	(0.011)	(0.011)
former USSR	−0.060 *	−0.060 ***	−0.061 *	−0.060 *	−0.070 **
	(0.032)	(0.013)	(0.032)	(0.032)	(0.035)
Anglo-Saxon/Liberal	0.198 ***	0.198 ***	0.196 ***	0.198 ***	0.180 ***
	(0.033)	(0.010)	(0.033)	(0.033)	(0.033)
Conservative/Corporatist	0.217 ***	0.217 ***	0.216 ***	0.217 ***	0.202 ***
	(0.034)	(0.007)	(0.034)	(0.034)	(0.033)
Social-democrat/Nordic	0.247 ***	0.247 ***	0.247 ***	0.250 ***	0.227 ***
	(0.032)	(0.012)	(0.033)	(0.033)	(0.033)
Constant	4.545 ***	4.545 **	4.300 **	4.226 **	
	(1.671)	(1.734)	(1.729)	(1.743)	
Observations	12,803	12,803	12,012	11,736	12,803
R-squared	0.157	0.157	0.151	0.153	

Note: All regressions include country dummies. Robust standard errors in parentheses. (1) main specification: linear probability model using robust standard errors, (2) main specification using clustered standard errors by country, (3) main specification without the two countries with mandatory vaccination (Bulgaria and the Czech Republic), (4) main specification without countries that recommend the flu vaccination for all individuals 55+ (Austria, Malta, Poland), and (5) main specification using a probit model. *** *p* < 0.01, ** *p* < 0.05, * *p* < 0.1.

**Table 4:** Information and attitudes on vaccination.

	Mean	Std. Dev.
QC11: A vaccination program establishes what vaccines a person should receive and at what time in life (like a calendar), as recommended by a health authority. At which level do you think vaccination programs should be coordinated?		
At international level	0.334	0.472
At European level	0.265	0.441
At national level	0.406	0.491
At regional or local level	0.165	0.371
There should be no vaccination programs, it is a personal choice	0.085	0.278
- post-communist	0.098	0.298
- former USSR	0.152	0.359
- Anglo-Saxon	0.031	0.175
- Conservative/Corporatist	0.092	0.289
- Social-democrat/Nordic	0.048	0.214
Don’t know	0.055	0.228
QC12: In the past six months, have you seen, read or heard any information on vaccination in the media?		
No	0.341	0.474
Yes, on TV	0.556	0.497
Yes, on the radio	0.148	0.355
Yes, in newspapers or magazines	0.197	0.398
Yes, on online social networks	0.037	0.188
Yes, on other Internet sites	0.044	0.206
Other (SPONTANEOUS)	0.013	0.113
Don’t know	0.014	0.118

Note: Multiple answers allowed. N = 12,803. Weighted means to represent the population 55+.

## Data Availability

Publicly available datasets were analyzed in this study. The data can be found here: [https://search.gesis.org/research_data/ZA7562 (accessed on 17 February 2021)].

## Supplementary Materials

The following are available online at https://www.mdpi.com/2076-393X/9/2/169/s1, Text 1, Table S1, Vaccination schedule (general population 55+).

## References

[1] European Centre for Disease Prevention and Control (ECDC) (2016). Let’s Talk about Protection.

[2] WHO (2019). Ten Threats to Global Health in 2019.

[3] European Centre for Disease Prevention and Control (ECDC) (2020). Vaccine Scheduler.

[4] Yaqub O., Castle-Clarke S., Sevdalis N., Chataway J. (2014). Attitudes to vaccination: A critical review. Soc. Sci. Med..

[5] Machado A., Mazagatos C., Dijkstra F., Kislaya I., Gherasim A., McDonald S.A., Kissling E., Valenciano M., Meijer A., Hooiveld M. (2019). Impact of influenza vaccination programmes among the elderly population on primary care, Portugal, Spain and The Netherlands: 2015/16 to 2017/18 influenza seasons. Eurosurveillance.

[6] European Centre for Disease Prevention and Control (ECDC) (2016). Let’s Talk about Hesitancy: Enhancing Confidence in Vaccination and Uptake.

[7] Ciabattini A., Nardini C., Santoro F., Garagnani P., Franceschi C., Medaglini D. (2018). Vaccination in the elderly: The challenge of immune changes with aging. Semin. Immunol..

[8] McLaughlin J.M., McGinnis J.J., Tan L., Mercatante A., Fortuna J. (2015). Estimated Human and Economic Burden of Four Major Adult Vaccine-Preventable Diseases in the United States, 2013. J. Prim. Prev..

[9] de Gomensoro E., Del Giudice G., Doherty T.M. (2018). Challenges in adult vaccination. Ann. Med..

[10] European Commission (2019). Special Eurobarometer 488: Europeans’ Attitudes towards Vaccination. https://dataeuropaeu/euodp/en/data/dataset/S2223_91_2_488_ENG.

[11] Andersen R., Davidson P. (2014). Improving access to care in America: Individual and contextual indicators. Changing the US Health Care System: Key Issues in Health Services Policy and Management.

[12] Miyachi T., Takita M., Senoo Y., Yamamoto K. (2020). Lower trust in national government links to no history of vaccination. Lancet.

[13] D’Ancona F., D’Amario C., Maraglino F., Rezza G., Iannazzo S. (2019). The law on compulsory vaccination in Italy: An update 2 years after the introduction. Eurosurveillance.

[14] Schmitz H., Wübker A. (2011). What determines influenza vaccination take-up of elderly Europeans?. Health Econ..

[15] Esping-Andersen G. (1990). The Three Worlds of Welfare Capitalism.

[16] Fenger M. (2007). Welfare regimes in Central and Eastern Europe: Incorporating post-communist countries in a welfare regime typology. Contemp. Issues Ideas Soc. Sci..

[17] ECDC (2018). Seasonal Influenza Vaccination and Antiviral Use in EU/EEA Member States. https://wwwecdceuropaeu/sites/default/files/documents/seasonal-influenza-antiviral-use-2018pdf.

[18] Loiacono M.M., Mahmud S.M., Chit A., van Aalst R., Kwong J.C., Mitsakakis N., Skinner L., Thommes E., Bricout H., Grootendorst P. (2020). Patient and practice level factors associated with seasonal influenza vaccine uptake among at-risk adults in England, 2011 to 2016: An age-stratified retrospective cohort study. Vaccine X.

[19] Sato A.P.S., Antunes J.L.F., Lima-Costa M.F.F., de Andrade F.B. (2020). Influenza vaccine uptake among older adults in Brazil: Socioeconomic equality and the role of preventive policies and public services. J. Infect. Public Health.

[20] Burns V.E., Ring C., Carroll D. (2005). Factors influencing influenza vaccination uptake in an elderly, community-based sample. Vaccine.

[21] Bertoldo G., Pesce A., Pepe A., Pelullo C.P., Di Giuseppe G., The Collaborative Working Group (2019). Seasonal influenza: Knowledge, attitude and vaccine uptake among adults with chronic conditions in Italy. PLoS ONE.

[22] Nagata J.M., Hernández-Ramos I., Kurup A.S., Albrecht D., Vivas-Torrealba C., Franco-Paredes C. (2013). Social determinants of health and seasonal influenza vaccination in adults ≥65 years: A systematic review of qualitative and quantitative data. BMC Public Health.

[23] Altpeter E., Wymann M.N., Richard J.-L., Mäusezahl-Feuz M. (2018). Marked increase in measles vaccination coverage among young adults in Switzerland: A campaign or cohort effect?. Int. J. Public Health.

[24] Rey D., Fressard L., Cortaredona S., Bocquier A., Gautier A., Peretti-Watel P., Verger P. (2018). Vaccine hesitancy in the French population in 2016, and its association with vaccine uptake and perceived vaccine risk–benefit balance. Eurosurveillance.

[25] Fall E., Izaute M., Chakroun-Baggioni N. (2018). How can the health belief model and self-determination theory predict both influenza vaccination and vaccination intention ? A longitudinal study among university students. Psychol. Health.

[26] Alhalaseh L., Fayoumi H., Khalil B. (2020). The Health Belief Model in predicting healthcare workers’ intention for influenza vaccine uptake in Jordan. Vaccine.

[27] Rosenstock I.M. (1974). Historical Origins of the Health Belief Model. Health Educ. Monogr..

[28] Janz N.K., Becker M.H. (1984). The Health Belief Model: A Decade Later. Health Educ. Q..

[29] Bertoncello C., Ferro A., Fonzo M., Zanovello S., Napoletano G., Russo F., Baldo V., Cocchio S. (2020). Socioeconomic Determinants in Vaccine Hesitancy and Vaccine Refusal in Italy. Vaccines.

[30] Mathieu P., Gautier A., Raude J., Goronflot T., Launay T., Debin M., Guerrisi C., Turbelin C., Hanslik T., Jestin C. (2019). Population perception of mandatory childhood vaccination programme before its implementation, France, 2017. Eurosurveillance.

[31] Endrich M.M., Blank P.R., Szucs T.D. (2009). Influenza vaccination uptake and socioeconomic determinants in 11 European countries. Vaccine.

[32] Fedson D.S., Hannoun C., Leese J., Sprenger M.J., Hampson A.W., Bro-Jørgensen K., Ahlbom A.M., Nøkleby H., Valle M., Olafsson O. (1995). Influenza vaccination in 18 developed countries, 1980–1992. Vaccine.

[33] Kunze U., Groman E., Böhm G., Kunze M. (2007). Influenza vaccination in Austria, 1982–2003. Wien. Med. Wochenschr..

[34] McHugh S.M., Browne J., O’Neill C., Kearney P.M. (2015). The influence of partial public reimbursement on vaccination uptake in the older population: A cross-sectional study. BMC Public Health.

[35] Blank P., Schwenkglenks M., Szucs T.D. (2012). The impact of European vaccination policies on seasonal influenza vaccination coverage rates in the elderly. Hum. Vaccines Immunother..

[36] Shemesh A.A., Rasooly I., Horowitz P., Lemberger J., Ben-Moshe Y., Kachal J., Danziger J., Clarfield A.M., Rosenberg E. (2008). Health behaviors and their determinants in multiethnic, active Israeli seniors. Arch. Gerontol. Geriatr..

[37] Farmanara N., Sherrard L., Dubé È., Gilbert N.L. (2018). Determinants of non-vaccination against seasonal influenza in Canadian adults: Findings from the 2015–2016 Influenza Immunization Coverage Survey. Can. J. Public Health.

[38] Bödeker B., Remschmidt C., Schmich P., Wichmann O. (2015). Why are older adults and individuals with underlying chronic diseases in Germany not vaccinated against flu? A population-based study. BMC Public Health.

