# Supplementary material for: Understanding the Influence of Individual and Systemic Factors on Vaccination Take-Up in European Citizens Aged 55 or Older
Source: Vaccines (Basel). 2021 Feb 17;9(2):169. doi: 10.3390/vaccines9020169 (PMC7922776; doi:10.3390/vaccines9020169)
Supplement: Supplementary file 1 [file vaccines-09-00169-s001.pdf]

Supplement

Text 1

In our analysis, we include both individual as well as systemic variables. Since we do not observe respondents' health, the classical need variable, we use official vaccination recommendations as indicator for the need of vaccination. Table S1 shows the vaccination schedule for the general population 55+ based on the ECDC vaccine scheduler [1]. Vaccination recommendations exist in all countries except Lithuania. By far the most frequent recommendation is the vaccination against influenza (27 countries), followed by the vaccinations against pneumococcal disease (18 countries), tetanus (14 countries), and diphtheria (12 countries). Only few countries recommend vaccinations against herpes zoster and pertussis (6 countries, each), poliomyelitis (3 countries), and tick-borne encephalitis (2 countries). The necessary doses for immunization differ largely between the different diseases. While in some cases only one dose is recommended (pneumococcal disease and herpes zoster), others generally require refreshers between five and 15 years (tetanus, diphtheria, pertussis, poliomyelitis, and tick-borne encephalitis) with somewhat more frequent refreshers recommended for older age groups. The influenza vaccination is an exception since it requires yearly vaccinations. A general recommendation for the vaccination exists for the population aged 55+ in three countries, for those aged 60+ in five countries, and for those aged 65+ in 19 countries.

Table S1: Vaccination schedule (general population 55+)

	Influenza[1]	Diphteria	Herpes Zoster	Pertussis	Pneumococcal Disease	Poliomyelitis	Tetanus	Tick-Borne Encephalitis
Austria	any age*	10 (>60: 5) years*	50+*	5 years*	50+*	10 (60+: 5) years*	10 (60+: 5) years*	5 (60+: 3) years*
Belgium	65+	10 years*	-	10 years*	65+	-	10 years*	-
Bulgaria	65+*	10 years[m]	-	-	-	-	10 years[m]	-
Croatia	65+	-	-	-	-	-	at 60[m]	-
Cyprus	65+	-	-	-	65+	-	-	-
Czech Republic	65+	-	50+*	10-15 years*	65+*	-	10-15 years[m]	5 (60+: 3) years*
Denmark	65+	-	-	-	65+*	-	-	-
Estonia	65+*	-	-	-	-	-	-	-
Finland	65+	-	-	-	65+*	-	-	-
France	65+	65+: 10 years	65-74	-	-	65+: 10 years	65+: 10 years	-
Germany	60+	10 years	at 60	10 years	60+	-	10 years	-
Greece	60+	10 years	60+	10 years	65+	10 years	10 years	-
Hungary	60+	-	-	-	50+*	-	-	-
Ireland	65+	-	-	-	65+	-	-	-
Italy	65+	10 years	65+	10 years	65+	-	10 years	-
Latvia	65+	10 years	-	-	-	-	10 years	-
Lithuania	-	-	-	-	-	-	-	-
Luxembourg	65+	-	-	-	65+*	-	-	-
Malta	55+	-	-	-	65+	-	-	-
Netherlands	60+	-	-	-	-	-	-	-
Poland	55+*	-	-	-	50+*	-	-	-
Portugal	65+	65+: 10 years	-	-	-	-	65+: 10 years	-
Romania	65+*	-	-	-	-	-	-	-
Slovakia	60+	15 years	-	-	-	-	15 years	-
Slovenia	65+	10 years	-	-	65+*	-	10 years	at 49
Spain	65+	at 65	-	-	65+	-	at 65	-
Sweden	65+	65+	-	-	-	-	-	-

United Kingdom	65+	-	70+	-	at 65	-	-	-

Note: *not funded; m: mandatory. Source: ECDC Vaccine Scheduler. The table shows the recommended vaccination after the age of 55 for the general population in different European countries as implemented in the year 2018, prior to the Eurobarometer survey. [1]Influenza vaccination is recommended annually.

References

1. European Centre for Disease Prevention and Control (ECDC). Vaccine Scheduler. 2020.
